# An Unusual Cause of Subarachnoid Hemorrhage

**DOI:** 10.1097/WOX.0b013e31818af155

**Published:** 2008-11-15

**Authors:** Yuen Su, Constance Katelaris

**Affiliations:** 1 Westmead Hospital, Sydney, New South Wales, Australia; 2 University of Western Sydney, Sydney, New South Wales, Australia; 3 Department of Medicine, Campbelltown Hospital, Therry Rd, Campbelltown, NSW Australia

**Keywords:** exercise-induced anaphylaxis, subarachnoid hemorrhage, food-dependent exercise-induced anaphylaxis

## Abstract

A 47-year-old man presented to our hospital with collapse secondary to a subarachnoid hemorrhage. A careful history taking revealed symptoms of anaphylaxis before his collapse. This case illustrates an unusual cause of subarachnoid hemorrhage.

## 

A 47-year-old man was brought in by an ambulance disoriented after a collapse. Neurological examination revealed mild right pyramidal upper limb weakness and flexor plantar reflex on the right.

The patient had been found by a passerby unconscious in a phone booth but regained consciousness by the time of ambulance arrival. He was noted to be hypotensive and flushed, and had a laceration over the right occiput. He was given oxygen en route to the hospital.

## Background history

The patient undertook regular exercise with brisk walking, which was always soon after his evening meal. Urticaria developed during some of his walks. Lip and chin paresthesia, tongue angioedema, and nausea were sometimes associated with these episodes. On a number of occasions, he had taken prednisolone 50 mg, prescribed by his general practitioner, after development of symptoms and always continued exercising.

At the time of his presentation, he recalled developing urticaria and lip paresthesia 15 minutes into his evening walk. He took 50 mg prednisolone and continued walking for a further half hour. He felt increasingly unwell and entered a phone booth to ring for help. He was unable to recall subsequent events.

The patient also had type 2 diabetes mellitus, well controlled with diet, and mild atopic rhinoconjunctivitis. He was on no regular medication.

## Investigations

Computer tomographic scan revealed a subarachnoid hemorrhage involving the left sylvian fissure and over the cerebral convexity. Cerebral angiogram and magnetic resonance imaging of the spine were normal. The etiology was presumed to be nonaneurysmal. A diagnosis of traumatic subarachnoid hemorrhage was made.

His clinical history was consistent with a diagnosis of exercise-induced anaphylaxis. A dietary history revealed that the ingestion of bread accompanied most of his evening meals.

Skin prick testing was performed with a range of inhalant and ingested allergens, including extracts of foods he commonly ate (Hollister-Stier Laboratories, Spokane, Wash) as well as with positive and negative controls (histamine and glycerosaline, respectively). He had positive skin test responses to grass pollens--rye grass (wheal diameter, 7 mm), timothy (14 mm), Bermuda (18 mm), and plantain (6 mm). Whole wheat extract gave a negative response, but a very strong positive reaction (9 mm) was seen with gluten.

Food-dependent exercise-induced anaphylaxis (EIA) was diagnosed. He has been advised to avoid gluten-containing products for at least 4 hours before exercising and has not experienced further episodes.

## Discussion

Subarachnoid hemorrhage has been uncommonly associated with nonconcussion-associated trauma [1].

Exercise-induced anaphylaxis is a clinical syndrome whereby anaphylaxis occurs in association with significant physical exertion [2]. Pruritus, urticaria, angioedema, and flushing are the most common presentations [3]. In some, food is an important cofactor in the triggering of attacks [4]. Food triggers may be specific or nonspecific [5]. The most common food precipitant is wheat. Gluten-free diet induced a complete remission of symptoms in 18 patients with wheat-associated food-dependent EIA [6]. Other identified food triggers include seafood, celery, alcohol, tomatoes, and nuts.

Emergency management of EIA is the same as that for anaphylaxis. Prevention of attacks includes food avoidance before exercise and education regarding the anaphylaxis prodrome so exercise can be ceased at the earliest warning signs. Patients should exercise with a partner, and self-injectable adrenaline should always be carried.

This case illustrates an unusual cause of subarachnoid hemorrhage in the context of head trauma as a result of collapse from anaphylaxis. Once exercise-induced anaphylaxis is diagnosed, detailed dietary history and skin testing with suspect foods may identify an essential cofactor in the etiology of the syndrome. In certain circumstances, cautious challenge tests involving exercise after ingestion of an implicated food maybe undertaken to confirm the association.
